# Improving the post-meal experience of hospitalised patients with eating disorders using visuospatial, verbal and somatic activities

**DOI:** 10.1186/s40337-016-0098-y

**Published:** 2016-03-11

**Authors:** Emily Griffiths, Nicholas Hawkes, Sam Gilbert, Lucy Serpell

**Affiliations:** University College London, Clinical, Educational & Health Psychology, 1-19 Torrington Place, London, WC1E 7HB UK; St Ann’s Eating Disorders Service, Barnet, Enfield and Haringey Mental Health NHS Trust, London, UK; University College London, Institute of Cognitive Neuroscience, 17 Queen Square, London, WC1N 3AR UK; Eating Disorders Service, North East London NHS Foundation Trust, London, UK

**Keywords:** Eating disorders, Affect, Post-meal tasks, Distraction

## Abstract

**Background:**

This study compares the effects of different cognitive tasks on post-meal negative affect, positive affect, intrusive thoughts and intrusive images of hospitalised patients with eating disorders.

**Methods:**

Twenty-five participants were recruited from an eating disorder service. Using a within-subjects design, participants performed one of the following tasks for 15 min: the game ‘Tetris’ (visuospatial), a general knowledge ‘Quiz’ (verbal), ‘Braille’ translation (somatic) and ‘Sitting Quietly’ (control). In total, participants completed each task on three occasions.

**Results:**

The visuospatial, verbal and somatic tasks had beneficial effects on all positive and negative indicators, when compared with ‘Sitting Quietly’. Visuospatial and somatic tasks were more effective at reducing intrusive imagery than the verbal task.

**Conclusions:**

The results suggest that certain engaging activities can help hospitalised patients with eating disorders manage the difficult post-meal period.

## Background

Mealtimes can be extremely distressing for individuals with eating disorders, resulting in symptoms of anxiety and depression [1–4]. This is likely to be heightened in eating disorder inpatients, who are frequently supervised and restricted in their activities following meals [5]. This study investigates whether visuospatial, verbal, and somatic activities performed in a hospital setting can reduce post-meal distress.

Inpatient eating disorder services vary widely in how they implement mealtimes [6], mostly relying on clinical judgement [7]. Although almost half of services assessed in the UK reported offering a post-meal activity [8] there is limited evidence to support the provision of specific activities during this time (though see [9–11]).

The underlying processes that underpin post-meal distress in people with eating disorders are not fully understood. People with eating disorders report feeling fat [12], which has been associated with distress, negative bodily images/sensations, and negative self-beliefs [13, 14]. One theory suggests that feeling fat results from a misinterpretation of particular emotions of depression, anxiety or guilt and bodily sensations of feeling full, bloated and sweaty [12, 15]. Considering this theory, it is possible that distress after mealtimes is a result of misinterpreting bodily sensations, such as the stomach stretching, which leads to a variety of images such as an overly expanded stomach. This is then viewed as evidence for the feared catastrophe: rapid weight gain. Therefore, one possible way to reduce distress during the post-meal period may involve interrupting the processing of feeling fat either through interrupting intrusive imagery, intrusive thoughts or somatic experiences. Studies within the Post-Traumatic Stress Disorder (PTSD) literature have considered a similar approach of interrupting intrusive imagery to reduce later traumatic flashbacks [16, 17].

The aim of this study is therefore to investigate the effects of post-meal visuospatial, verbal and somatic tasks on self-reported positive and negative affect and intrusive thoughts and intrusive imagery, in comparison with a control condition of sitting quietly. This may help to shed light on suitable activities to improve the difficult post-meal experience.

## Methods

### Participants

Participants were recruited from three hospital units within the same eating disorders service: an inpatient ward, a residential rehabilitation unit and a day hospital. Participants were required to be between 18-65 years old and were excluded if they were unable to speak English fluently, had a moderate to severe learning disability, were on bed-rest or were imminently being discharged.

### Procedure

This study used a within-subjects design. Immediately following their meals (breakfast, lunch or dinner) whilst on the unit, participants accessed an online web-based programme using a laptop. This programme administered various questionnaires (see below), and one of four tasks, which lasted for 15 min.

Participants on the ward or rehabilitation unit repeated this procedure following any 12 meals (breakfast, lunch or dinner) of their choice, which they completed within a two week (inpatients) or one month (day patients) period. Each of the four tasks was assigned to three of the sessions in a Latin Square design so that participants could not predict in advance which task they would perform, and each task was equally likely to occur at each of the 12 time points.

### Tasks

1. Visuospatial (Tetris). A computerised game requiring participants to use cursor keys to rotate falling geometric blocks (see “http://www.ucl.ac.uk/sam-gilbert/tetris.php”).
2. Verbal (Quiz). A computerised general knowledge quiz requiring participants to select answers from four possible choices (see “http://www.ucl.ac.uk/sam-gilbert/quiz.php”).
3. Somatic (Braille). A novel task requiring participants to use their fingertips to translate a random list of Braille letters into letters of the English alphabet.
4. Control (Sitting Quietly). Participants were required to sit quietly.

### Measures

The Positive and Negative Affect Scale (PANAS; [18]) consists of 10 items of positive affect and 10 items of negative affect, each of which is rated on a scale from 1 (very slightly/ not at all) to 5 (extremely). The measure has been validated among adult samples, with alpha coefficients ranging from 0.84-0.90 [18]. This scale was administered at the beginning of each session (before the participant knew which task they would perform) and again immediately following the task. Any short-term effect of the task during the post-meal period was measured by the change score, where a positive change score on the PANAS negative affect subscale would represent an increase in negative affect over time and a positive change score on the PANAS positive affect subscale would represent an increase in positive affect over time.

Participants were also asked the following two questions after the task had been administered; ‘To what extent did you experience intrusive body and fatness related thoughts during the activity?’ and ‘To what extent did you experience intrusive body and fatness related images during the activity?’ These questions were rated from 1 (very slightly/not at all) to 5 (extremely).

### Ethical approval

Ethical approval was granted from the local NHS Research Ethics Committee. Informed consent to participate was obtained from all participants.

## Results

**Fig. 1:**
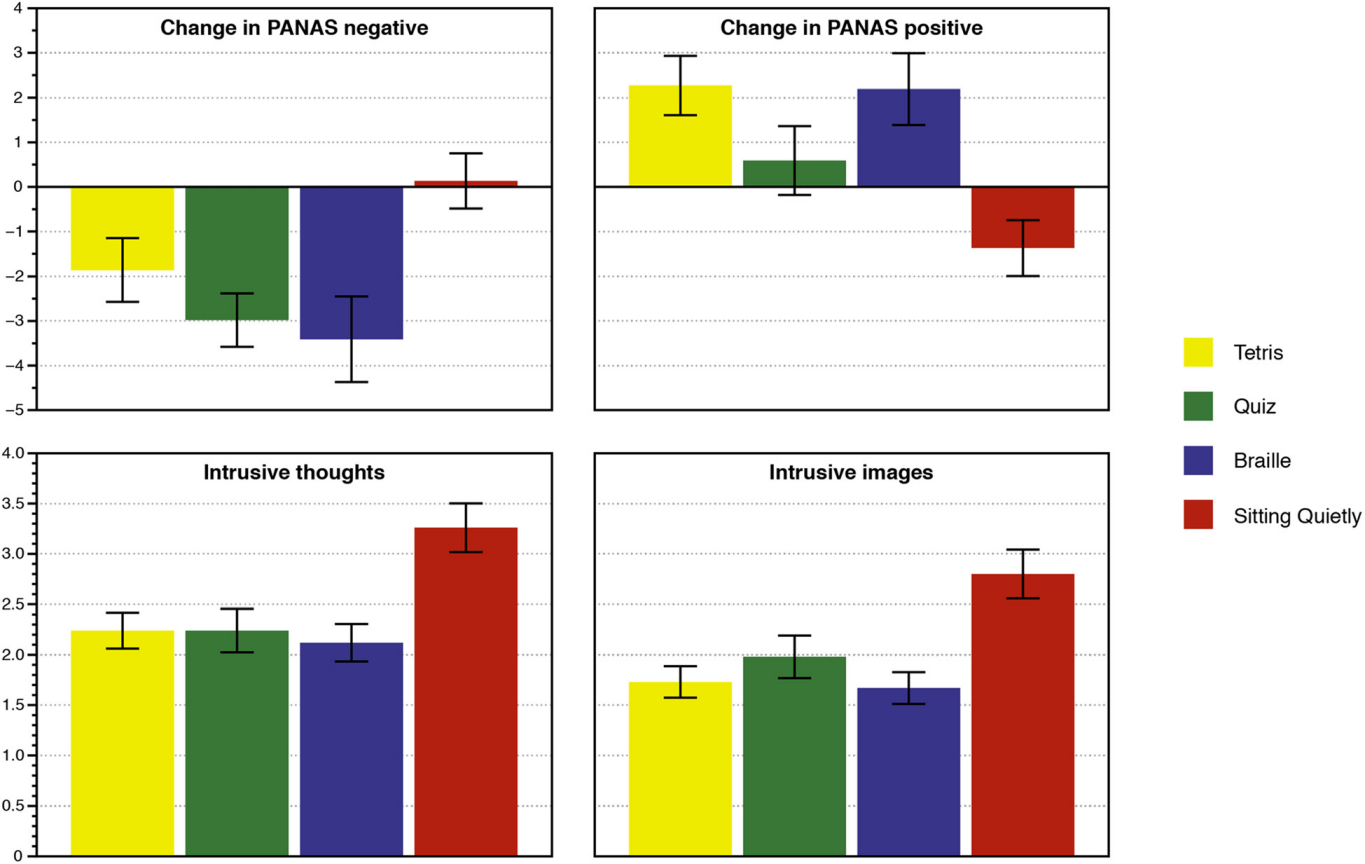
Changes in PANAS scores, intrusive thoughts and intrusive images following post-meal activities

Of those suitable, 36 (69 %) agreed to take part in the study. Subsequently 11 participants dropped out, defined as completing fewer than 75 % of trials, versus 25 ‘completers’, who were included in subsequent analyses. Participants who dropped out of the study were twice as likely to be White British as completers. No other significant difference was observed between these two groups (see Table 1 for full clinical and demographic data). Outcome measures are shown in Fig. 1.

**Table 1:** Demographic and clinical data

Demographic	Completers (*n* = 25)	Drop Outs (*n* = 11)	*p* value
Unit (%)			
Inpatient	52.0	90.9	
Rehabilitation	32.0	0	.06^a^
Day Hospital	16.0	9.1	
Age, years: mean (S.D.)	27.4 (9.8)	36.7 (15.0)	.12^b^
Gender: % F	96.0	100	.50 ^a^
Ethnicity (% WB)	36.0	72.7	.04 ^a^
Employment status (%)			
Unemployed	16.0	63.6	
Student	44.0	27.3	
Non-professional/voluntary	16.0	9.1	.06 ^a^
Professional	20.0	0	
Retired	4.0	0	
Diagnosis (%)			
AN-R	64.0	72.7	
AN-BP	24.0	18.2	.88^a^
EDNOS	12.0	9.1	
BMI on admission: mean (S.D.)	15.4 (2.4)	13.9 (1.3)	.08^c^
Duration of illness, years: mean (S.D.)	9.9 (10.4)	20.3 (14.6)	.06^b^
Length of current stay in hospital^d^, days: mean (S.D.)	32.2 (49.3)	79.0 (140.9)	.22 ^b^
PHQ^e^: mean (S.D.)	17.0 (8.0)	17.1 (7.5)	.97^c^
GAD^f^: mean (S.D.)	13.2 (5.0)	13.8 (5.0)	.74 ^c^
EDE-Q^g^: mean (S.D.)			
Restraint	3.7 (2.0)	2.8 (2.6)	.32 ^c^
Eating Concern	3.6 (1.6)	3.0 (1.7)	.39 ^c^
Shape Concern	4.4 (1.5)	3.8 (0.9)	.30 ^c^
Weight Concern	3.9 (1.6)	3.5 (1.3)	.43 ^c^
Global	3.8 (1.5)	3.3 (1.3)	.31^c^

Note. *F* female, *WB* White British, *AN-R* Anorexia Nervosa, Restrictive subtype, *AN-BP* Anorexia Nervosa, Binge Purge subtype, *EDNOS* Eating Disorders Not Otherwise Specified, *BMI* Body Mass Index

^a^ Categorical data was compared using Chi-squared statistical test. ^b^ Data not meeting the assumptions for parametric tests was compared using Mann-Whitney U test. ^c^ Data meeting the assumptions for parametric tests was compared using independent samples *t*-test. ^d^ Length of current stay calculated from admission into hospital until commencing the study. ^e^ Patient Health Questionnaire [19]. ^f^ General Anxiety Disorder assessment [20]. ^g^ Eating Disorders Examination Questionnaire [21]

### PANAS

PANAS negative baseline scores did not differ significantly between tasks (Tetris: 23.6; Quiz: 23.9; Braille: 23.5; Sitting Quietly: 24.6; *p* = .53) but there was a significant effect of task on change scores (*p* = .002). PANAS negative was reduced from pre- to post-task in the Tetris, Quiz, and Braille conditions (*p* < .03), but not in the Sitting Quietly condition (*p* = .90). Pairwise comparisons revealed a significant difference between Sitting Quietly and: Quiz (*p* = .001) and Braille (*p* = .011), and a trend difference for Tetris (*p* = .087). There was also a difference between Tetris and Quiz (*p* = .023), with the Quiz leading to greater reduction in negative affect. No other significant differences were observed.

PANAS positive baseline scores also did not differ significantly between tasks (Tetris: 16.3; Quiz: 16.7; Braille: 16.2; Sitting Quietly: 16.2; *p* = .73) but again there was a significant effect of task on change scores (*p* = .005). PANAS positive was increased from pre- to post-task in the Tetris and Braille conditions (*p* < .01), did not change significantly in the Quiz condition (*p* = .67), and decreased in the Sitting Quietly condition (*p* = .04). Pairwise comparisons revealed a significant difference between Sitting Quietly and: Tetris (*p* = .001) and Braille (*p* = .006). There was also a difference between Quiz and Tetris (*p* = .014). No other significant differences were observed.

### Intrusive thoughts and images

There was a significant effect of task on ratings of both intrusive thoughts and images (*p* < .001). For both ratings, pairwise comparisons between Sitting Quietly and each of the three other tasks were significant (*p* < .002). Compared with Quiz, there was a significant reduction in intrusive images for Braille (*p* = .033) and a trend for Tetris (*p* = .08). Ratings of intrusive thoughts did not differ significantly between the three active tasks.

## Discussion

The findings of this study show that engaging in an activity after meals helps to improve the post-meal experience for hospitalised patients with eating disorders. Compared with sitting quietly, all three tasks increased positive affect, decreased negative affect, and decreased intrusive thoughts and images. There was also evidence for nonequivalence between tasks: compared with the Quiz task, Braille was significantly more effective at reducing intrusive imagery and Tetris led to significantly more favourable PANAS positive. However, Quiz was significantly more effective than Tetris for reducing PANAS negative. Thus there is preliminary evidence that post-meal distress may be multidimensional and might be addressed by specific mechanisms beyond general distraction.

The main limitation of this study was that there were key differences between the three units used to obtain participants. They differed in terms of participant illness severity, how much participants were supervised following meals, levels of distraction and whether they could be on their own or not to complete the study. Completers and drop-outs differed significantly in ethnicity and there were marginally significant differences in other measures. In addition, the sample size was relatively small and participants varied in demographic factors and clinical indicators, creating a rather heterogeneous subject group. However, this heterogeneity might suggest that the highly significant effects observed are unlikely to depend critically on small procedural details or highly restricted participant demographics. Studies with more homogenous samples may help detect mediating factors for different cognitive tasks. A further question for future research is to investigate the maintenance over time of the effects reported in this article. It is acknowledged that the high drop-out rate from the study may represent a challenge in using this as an intervention in treatment settings.

## Conclusions

These findings add to the scarce literature providing evidence of the benefit of post-meal activities for eating disorders, and are of practical use to patients and supporting services that incorporate mealtimes, in particular hospitals. Improving patients’ ability to manage this time has the potential of improving their engagement with hospital treatment and improving clinical outcomes.
